# N6-Methyladenine DNA modification in *Xanthomonas oryzae* pv. *oryzicola* genome

**DOI:** 10.1038/s41598-018-34559-5

**Published:** 2018-11-02

**Authors:** Chuan-Le Xiao, Shang-Qian Xie, Qing-Biao Xie, Zhao-Yu Liu, Jian-Feng Xing, Kai-Kai Ji, Jun Tao, Liang-Ying Dai, Feng Luo

**Affiliations:** 1 Southern Regional Collaborative Innovation Center for Grain and Oil Crops in China, College of Plant Protection, Hunan Agricultural University, Changsha, China; 2 Hainan Key Laboratory for Sustainable Utilization of Tropical Bioresources, Institute of Tropical Agriculture and Forestry, Hainan University, Haikou, 570228 China; 3 School of Computing, Clemson University, Clemson, 29634-0974 USA

## Abstract

DNA N6-methyladenine (6mA) modifications expand the information capacity of DNA and have long been known to exist in bacterial genomes. *Xanthomonas oryzae* pv. *Oryzicola* (*Xoc*) is the causative agent of bacterial leaf streak, an emerging and destructive disease in rice worldwide. However, the genome-wide distribution patterns and potential functions of 6mA in *Xoc* are largely unknown. In this study, we analyzed the levels and global distribution patterns of 6mA modification in genomic DNA of seven *Xoc* strains (BLS256, BLS279, CFBP2286, CFBP7331, CFBP7341, L8 and RS105). The 6mA modification was found to be widely distributed across the seven *Xoc* genomes, accounting for percent of 3.80, 3.10, 3.70, 4.20, 3.40, 2.10, and 3.10 of the total adenines in BLS256, BLS279, CFBP2286, CFBP7331, CFBP7341, L8, and RS105, respectively. Notably, more than 82% of 6mA sites were located within gene bodies in all seven strains. Two specific motifs for 6 mA modification, ARGT and AVCG, were prevalent in all seven strains. Comparison of putative DNA methylation motifs from the seven strains reveals that *Xoc* have a specific DNA methylation system. Furthermore, the 6 mA modification of *rpfC* dramatically decreased during *Xoc* infection indicates the important role for *Xoc* adaption to environment.

## Introduction

DNA methylation, a base modification, does not alter the underlying DNA sequence, but adds additional information to bases through the addition of a methyl group. A group of enzymes, called DNA methyltransferases, catalyze the methylation process, and the methylated bases are assigned a name that reflects the atom harboring the methyl group^1,2^. Methylation on the fifth position of the pyrimidine ring of cytosine (5-methylcytosine, 5mC) is the predominant DNA methylation modification in eukaryotes^3,4^. The existence and abundance of a methyl group at the sixth position of the purine ring of adenine (N6-methyldeoxyadenosine, 6mA) was firstly reported in eukaryotes^3,5^. But several studies showed that the 6 mA base was present at extremely low levels in genomic DNA of higher eukaryotes^6^. The development of high throughput sequencing has greatly promoted the research and identification of 6 mA in fungi, plants, animals and humans^4,7–12^, which can further reveal the genome-wide distribution patterns of 6 mA modifications as well as different functions in biological processes among organisms. 6 mA modification was found as a prevalent DNA methylation in prokaryotes, which is used as a signal for epigenetic regulation^13^.

DNA 6 mA modification is ubiquitous in microbial genomes and plays an important role in regulating the biological processes in bacteria. It discriminates the host DNA from foreign pathogenic DNA and protects the host genome via the restriction-modification system, associated with defense against bacteriophages^14,15^. In addition, 6 mA is also involved in bacterial DNA replication and repair^16,17^, cell-cycle progression, and gene regulation^15,18,19^. Recently, the genome-wide distribution patterns of 6 mA have been investigated extensively using single molecule real-time (SMRT) sequencing^20^, which allows genome-wide mapping of m6A in bacteria at single-nucleotide resolution and at single-molecule level.

Single molecule real-time (SMRT) sequencing has been used for identification of 6 mA modifications in several bacterial genera, including *Helicobacter pylori*, *Lactobacillus* spp., *Mycobacterium tuberculosis*, *Escherichia coli*, *Campylobacter coli* and *Xanthomonas* spp.^21–25^. Among them, *Xanthomonas* spp. is the only agricultural genus for methylation detection. *Xanthomonas* causes serious and devastating diseases in more than 400 plants, including important economic and agricultural crops such as tomato, pepper, soybean and rice^26^. Bacterial leaf streak in rice, a devastating diseases in the world, is caused by *Xanthomonas oryzae* pv. *oryzicola* (*Xoc*)^27^. However, the genome-wide distribution patterns and potential functions of 6 mA in *Xoc* are still unknown. In this study, we measured the levels and global distribution patterns of 6 mA in genomic DNA of seven *Xoc* strains (BLS256, BLS279, CFBP2286, CFBP7331, CFBP7341, L8 and RS105), and compared DNA methylation motifs among seven strains.

## Materials and Methods

### Identification of 6 mA in *Xoc* genome

The raw data files of SMRT sequencing reads in h5 format were downloaded from the NCBI SRA database (Table S1)^28^. For each strain, 4–7 SMRT cells were used to achieve ~200 × coverage (Table S1). All cells used the P4C2 chemistry. Then, PacBio SMRT analysis platform (version 2.3.0) was used to detect DNA 6 mA modification of each strain (http://www.pacb.com/products-and-services/analytical-software/smrt-analysis/analysis-applications/epigenetics/). The detailed analysis workflow is as follows: Firstly, the raw reads were aligned to the corresponding reference genome of each strain by pbalign with the parameters ‘–seed = 1 –minAccuracy = 0.75 –minLength = 50 –concordant –algorithmOptions = “-useQuality” –algorithmOptions = ‘ -minMatch 12 -bestn 10 -minPctIdentity 70.0” (the reference resources are listed in Table S1). Furthermore, the polymerase kinetics information was loaded after alignment by loadChemistry.py and loadPulses scripts of raw h5 format files with‘-metrics DeletionQV, IPD, InsertionQV, PulseWidth, QualityValue, MergeQV, SubstitutionQV, DeletionTag’. The post-aligned datasets were sorted by using cmph5tools. The m6A was identified by using ipdSummary.py script with‘–methylFraction –identify m6A, m4C –numWorkers 4′. 6 mA sites with less than 25-fold coverage per chromosome of each strain were excluded for further analysis.

### Bioinformatics analysis

The genome-wide 6 mA profiles across all chromosomes of seven *Xoc* strains were generated using Circos^29^. The gene bodies, intergenic regions and translation stop codons were defined by using an annotated file (gff format) of each strain by using in-house shell scripts. For each 6 mA modification site, we extracted 20 bp from the upstream and downstream sequences of the 6 mA site. The MEME was then used to predict conserved motifs in the flanking regions^30^.

### 6 mA levels of *Xoc* virulence-related genes during infection

Twenty-five-day-old leaves from the rice cultivar TP309 were inoculated with Xoc RS105 using the injection method. Bacteria for inoculation were taken from PSA plates and re-suspended in water at an OD_600_ of 0.3. 10 μl bacteria were injected into one rice leaf and 15 replicates were performed. Then the leaves were cut and DNA was isolated. The 6 mA levels of some virulence-related genes were analyzed according to previously described methods^31^.

## Results

### 6 mA overview in *Xoc* genome

**Figure 1:**
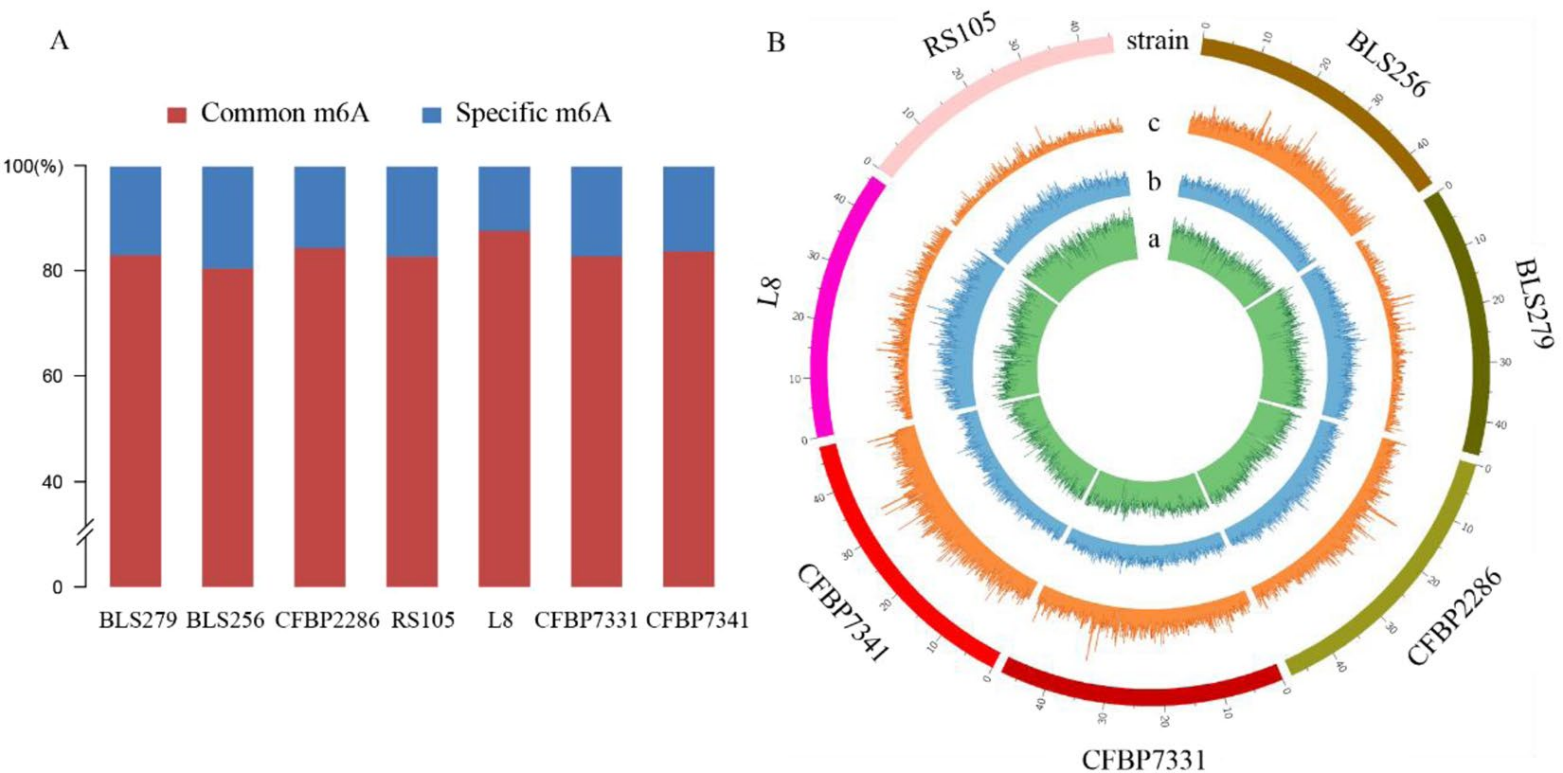
Distribution of 6 mA in seven Xoc strains. (**A**) Common and specific m6A sites; (**B**) circus plot of 6 mA in Xoc genome. a, density of 6 mA with fraction 0–0.3; b, density of 6 mA with fraction 0.3–0.7; c, density of 6 mA with fraction 0.7–1).

Analyzing SMRT sequencing datasets from *Xoc* genomic DNA, we detected 32751, 26395, 32779, 38009, 30694, 17900 and 26460 DNA 6 mA methylation sites in seven strains BLS256, BLS279, CFBP2286, CFBP7331, CFBP7341, L8 and RS105, respectively (Table 1 and Supplementary 1.xlsx). The density (6 mA/A) is about 2.1% to 4.2% of the total adenines in the *Xoc* genomic DNA (Table 1), which was close to the strain Xcv 85–10 of *Xanthomonas campestris* pv. *vesicatoria* (3.84%)^25^ and the *Hesseltinella vesiculosa* (2.8%)^12^,but higher than those in *Caenorhabditis elegans* (~0.7%)^8^, *Chlamydomonas* (~0.4%)^7^, *Drosophila* (0.07%)^9^, Human (0.051%) and *Arabidopsis thaliana* (0.048%)^4^. Among all of the 6 mA modification sites in the seven strains, nearly one-fifth was unique in each strain, and a large number of 6 mA sites (>80%) were identified in at least two strains (Fig. 1A and Supplementary 1.xlsx).

**Table 1:** Statistical overview of 6 mA modification in genomic DNA of seven *Xanthomonas oryzae* pv. *oryzicola*strains.

Strain	Genome size (Mb)	Total A number	m6A number	m6A ratio	Specific m6A	Common m6A
BLS256	4.608	868176	32751	3.80%	6354	26397
BLS279	4.569	864106	26395	3.10%	4459	21936
CFBP2286	4.774	894661	32779	3.70%	5093	27686
CFBP7331	4.776	905931	38009	4.20%	6496	31513
CFBP7341	4.785	905944	30694	3.40%	4954	25740
L8	4.574	865646	17900	2.10%	2196	15704
RS105	4.558	862859	26460	3.10%	4546	21914

The distribution and modification level of 6 mA was presented in circos plot format in which concentric rings represent the density distribution of 6 mA across all seven strains in the given category. Densities were divided into three categories, namely, low (0–30%, green circle), middle (30–70%, blue circle) and high (70–100%, orange circle). The 6 mA density in low modification level group was dominant in all strains (Fig. 1B).

### Analysis of 6 mA-methylated genes

**Figure 2:**
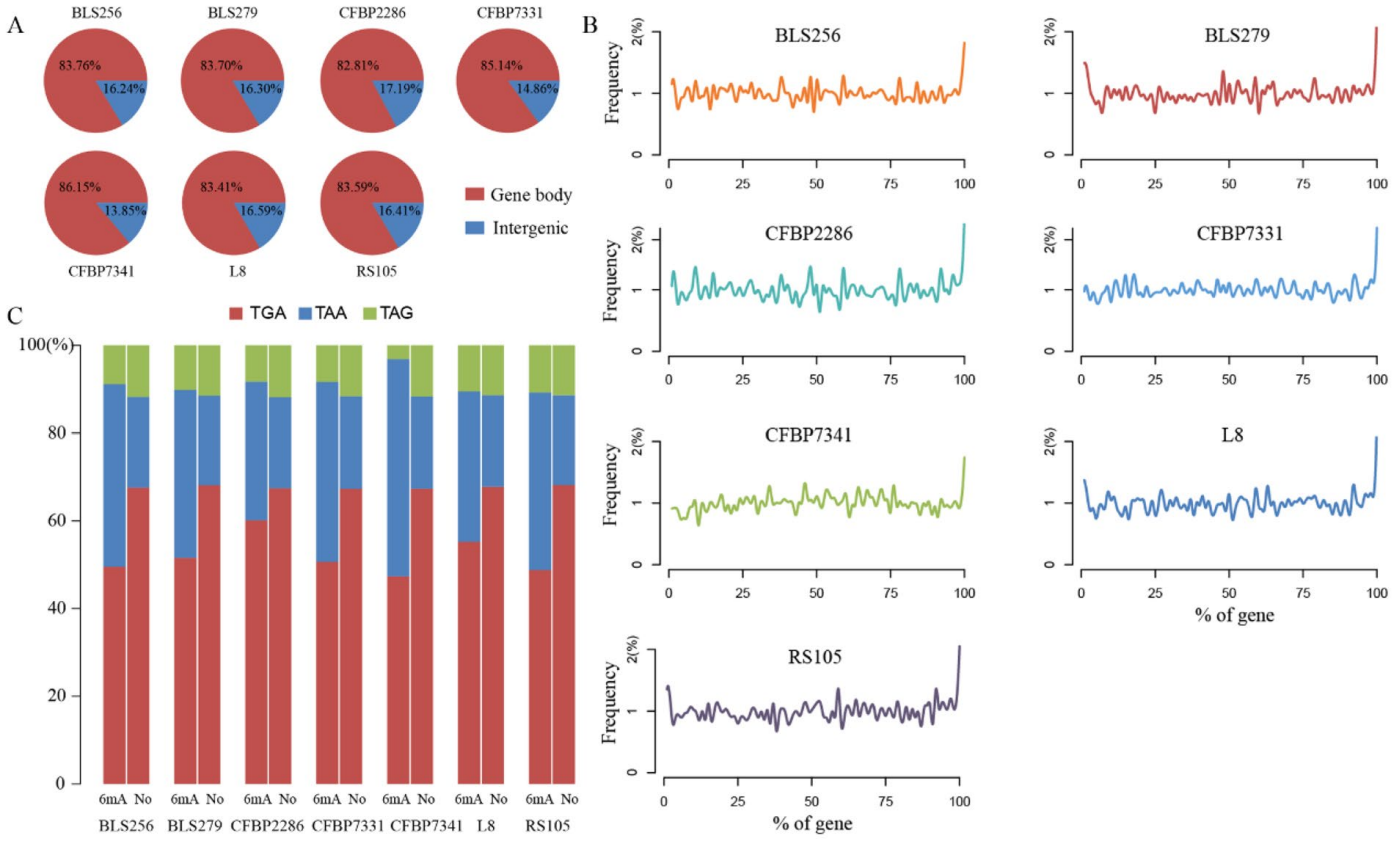
6 mA enrichment analysis in methylated genes. (**A**) Distribution of 6 mA sites in gene bodies and intergenic regions in seven strains; (**B**) frequency of 6 mA at relative position of protein coding genes; (**C**) stop codon usage in 6mA-methylated genes and no methylation genes)

We further analyzed the gene bodies and intergenic regions to examine 6 mA distribution around functional elements according to the annotated genome, and found that more than 82% of 6 mA sites were located in the gene bodies (Fig. 2A). Furthermore, while 82–93% of genes were methylated, the 6 mA methylation ratios of protein coding genes were higher than all methylated genes (Table 2). In all gene bodies, more than one 6 mA site was identified throughout the majority methylated genes (Fig. S1), and the number of 6 mA sites was associated with the gene length (Fig. S2). As the gene length increases, there is a tendency for increased number of methylation sites.

**Table 2:** The m6A methylation ratio in all and protein coding genes.

Strains	All			Protein coding		
	Total no.	m6A no.	Ratio	Total no.	m6A no.	Ratio
BLS256	4493	4162	92.63%	4267	4024	94.31%
BLS279	4485	3944	87.94%	4261	3886	91.20%
CFBP2286	4709	4253	90.32%	4482	4180	93.26%
CFBP7331	4711	4343	92.19%	4488	4276	95.28%
CFBP7341	4716	4227	89.63%	4493	4174	92.90%
L8	4479	3677	82.09%	4254	3631	85.35%
RS105	4480	3977	88.77%	4255	3916	92.03%

To further investigate the 6 mA locations in protein coding genes, the 6 mA relative distance in these genes were analyzed. The 6 mA sites were enriched at 3′ end of coding regions (Fig. 2B) which was different from those in human. Based on the annotated genome, these enriched locations were the terminal of coding regions where the stop codon usage were TAG, TAA and TGA. TGA was the stop codon detected in highest proportion in all of the analyzed sites, both modified and non-modified, detected at 50% and 68%, respectively. (Fig. 2C and Table S2). Notably, the proportion of TAA in 6 mA predicted sites were higher than that in normal codons, whereas TAG and TGA levels were reduced (Fig. 2C).

### Identification of consensus motifs for 6 mA in *Xoc*

**Figure 3:**
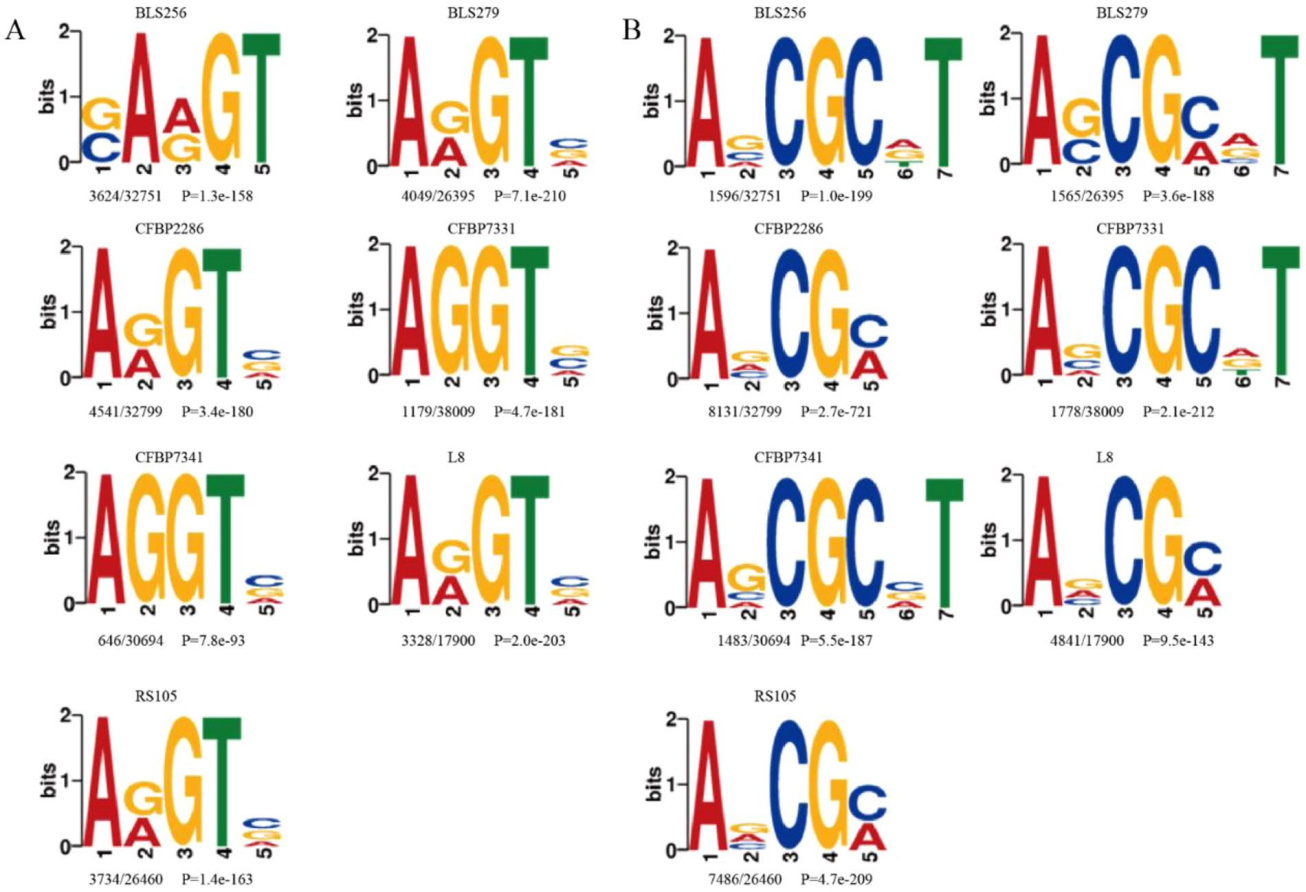
The identified consensus motifs containing 6 mA sites in seven strains. The number of occurrences of each motif relative to the total number of 6mA-containing motifs and the corresponding *p*-value generated by MEME are shown under the sequence logo.

To determine whether the identified 6 mA sites share consensus sequence element(s) in seven strains, we extracted the upstream and downstream 20 bp sequences from the 6 mA sites, and performed a default search for significant consensus motifs enriched in these regions using MEME. There were three significantly enriched motifs sequences AGG, ARGT and AVCG in all seven strains (Figs 3 and S3). The sequences AGG detected in *Xoc* was consistent with the motif sequences in *C. elegans*, *A. thaliana* and human. The most significant strain containing AGG sequences was CFBP7341 (p = 2.8e-1884), which was present in approximately one-fourth of the methylated sites (Fig. S3). Interestingly, besides AGG, two novel enriched DNA methylation motifs (ARGT and AVCG) were identified in all seven *Xoc* strains (Fig. 3). The p-values of motifs in all strains were less than 7.8e-93, which indicated the identified motifs are indeed prevalent in 6 mA sites.

### 6mA might be important for *Xoc* infection

**Figure 4:**
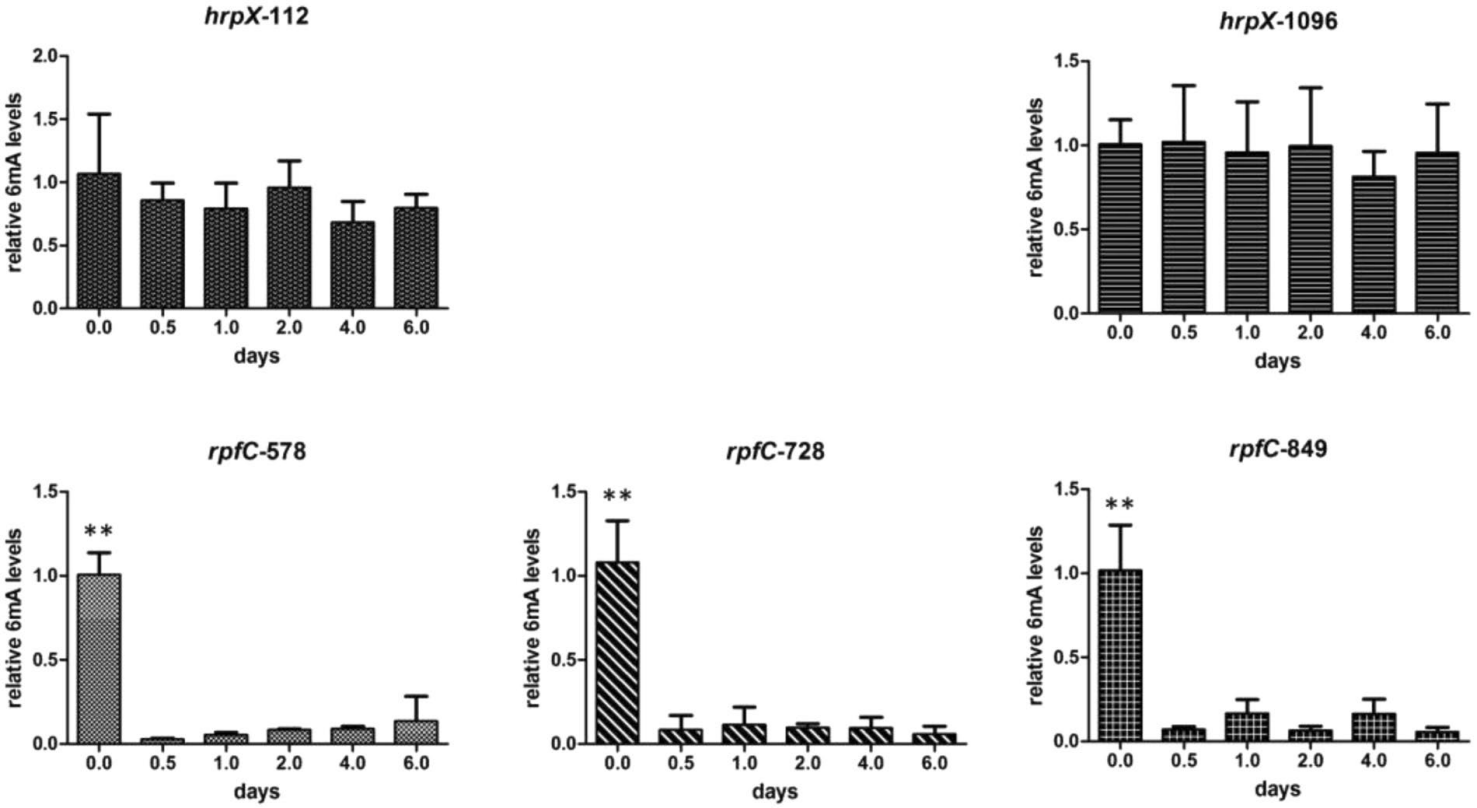
6 mA levels of special methylation sites in *rpfC* and *hrpX* during *Xoc* infection. The methylation levels at 0 days (DNA isolation immediately injection) were set as 1, and others were compared.

*Xoc* enters the host through the stomata or physical wounds and multiplies in the vascular regions. Its virulence is regulated by different mechanisms, but the pathways regulated by RpfC and HrpX are essential to successful infection^32,33^. Moreover, *rpfC* and *hrpX* have 6 mA sites (Table 3). Therefore, we chose these two genes to study the 6 mA levels during infection. As shown in Fig. 4, the methylation levels of three 6 mA sites in *rpfC* decreased significantly when growth in host (p < 0.01), but those in *hrpX* were not significantly changed, indicating that the 6 mA modification of *rpfC* is involved in *Xoc* adaptation to host environment. As RpfC is involved in quorum sensing and regulates the extracellular polysaccharide (EPS) levels, extracellular enzyme activity and motility in *Xanthomonas oryzae*^32,34^; However, HrpX regulates the expression of genes encoding the type-III secretion system which directly transports bacterial effectors into rice cells and regulates their functions^33,35^. These results imply that 6 mA might be important for *Xoc* adaptation to environment not for *Xoc*-rice direct interaction.

**Table 3:** The m6A methylation sites in *rpfC* and *hrpX*.

genes	6mA site	position	strand
*hrpX*	CGCAGAGATCGCTGCAAAGT**A**GGTCGAAAGGATCATGCCGG	112	−
*hrpX*	ACAAGCCTTGTTGCTCTACA**A**CCGCTATGCGCTGGACGCGG	1096	+
*rpfC*	ATTCGTGACTCATATTGGCC**A**GGAAACGGCTCTTGGCCTGG	578	−
*rpfC*	TCTGCTGTCGCTGGTGGAAG**A**GGTGCTGGATATTTCCGCGA	728	+
*rpfC*	CCGCAGGCCAGGACGCGCGG**A**CTGGATTACGGCACCGAGGT	849	+

## Discussion

N6-methyladenine (6 mA) is mainly found in prokaryotic genomes. The atlas of m6A modification sites have been elucidated in several bacteria strains by using SMRT sequencing, which opened up a new direction for epigenetics research^22–25^. In this paper, we studied the genome-wide distribution of DNA 6 mA modification among in seven *Xoc* strains, the pathogen responsible for bacterial leaf streak disease in rice. We found that that 6 mA sites are widely distributed in the *Xoc* genome and are enriched in gene bodies. Two previously unreported motifs, ARGT and AVCG, are involved in 6 mA modification among seven strains, which helps to understand 6 mA distribution patterns in *Xoc*.

SMRT sequencing datasets of seven *Xoc* strains were obtained from the NCBI SRA database^28^. We observed that 6 mA methylation density was similar in each strain (2.1–4.2%), and similar to the previously reported in bacterial genomes (1.9% in *Escherichia coli*, 2.7% in *Campylobacter coli* and 0.17–3.8% in *Xanthomonas campestris)*^22,24,25^. But our results were relatively higher than the density reported in *C. elegans* (~0.7%)^8^, *Chlamydomonas* (~0.4%)^7^, *Drosophila* (0.07%)^9^, Human (0.051%) and *A. thaliana* (0.048%). Interestingly, prokaryotic genomes have generally higher 6 mA levels than eukaryotes, which may be related to the defense mechanism of bacteriophage infection^36,37^. Adenine and cytosine methylation of bacterial DNA protects it from the action of the corresponding restriction endonuclease, whereas unmethylated sites of foreign nucleic acids, such as bacteriophage DNA are cleaved.

Sequence motifs are short recurring nucleotide sequences present throughout the genome. One 6 mA genomic distribution with the prevalent motif sequence AGG, similar to that in *C. elegans*, *A. thaliana* and human^4,8^, was detected in seven *Xoc* strains. Previous studies showed that the AGG motif sequence was widespread in eukaryotic species, and the motif GATC was likely the most ancient 6 mA motif exists in bacteria^22,24,25^. In this study, we find that the motif AGG, that was previously unreported in bacteria, was consistently present in all seven *Xoc* strains analyzed here (Fig. S3). Moreover, the motif GATC that consistent with previous reports were detected in the partial *Xoc* strains (Fig. S4). Importantly and interestingly, two specific motifs ARGT and AVCG were identified in all seven *Xoc* strains, which have not been reported in other organisms. These results indicate that the 6 mA modification pattern was not conserved across the species genome and is not species-specific, likely reflecting the potential diverse biological functions.

*Xoc* is an extracellular pathogen and infects rice through the stomata or physical wounds and grows in the vascular regions. The type II, III, VI secretion systems, quorum sensing and motility are all important for *Xoc*-rice interaction. *RpfC* and *HrpX* are two key regulators of *Xoc* virulence^32,33^. *RpfC* is involved in the regulation of quorum sensing, motility and Type II secretion system^32^, but *HrpX* regulates Type III secretion system that directly determines the *Xoc*-host interaction^33^. Therefore, the identified *rpfC* and *hrpX* 6 mA modification might affect *Xoc* adaption to environment and direct interaction with rice. The different changes of 6 mA modification levels in *rpfC* and *hrpX* indicate that the 6 mA modification in Xoc is associated with environmental adaptation.

In summary, we found that 6 mA was widely present in *Xoc* genomic DNA and detected two specific epidemic motifs, ARGT and AVCG, which promoted 6 mA modification. This study suggests that the 6 mA modification in *Xoc* is associated with environmental adaptation.

## Electronic supplementary material

Supplementary file

Supplementary dataset
