# Supplementary material for: N6-Methyladenine DNA modification in Xanthomonas oryzae pv. oryzicola genome
Source: Sci Rep. 2018 Nov 2;8:16272. doi: 10.1038/s41598-018-34559-5 (PMC6215013; doi:10.1038/s41598-018-34559-5)
Supplement: Supplementary file 1 — Supplementary file [file 41598_2018_34559_MOESM1_ESM.pdf]

N6-Methyladenine DNA modification in *Xanthomonas oryzae* pv. *oryzicola* genome

Chuan-Le Xiao[1, #], Shang-Qian Xie[2, #], Qing-Biao Xie[2], Zhao-Yu Liu[2], Jian-Feng Xing[2], Kai-Kai Ji[2], Jun Tao[2, *], Liang-Ying Dai[1, *], Feng Luo[1, 3, *]

[1] Southern Regional Collaborative Innovation Center for Grain and Oil Crops in China, College of Plant Protection, Hunan Agricultural University, Changsha, China

[2] Hainan Key Laboratory for Sustainable Utilization of Tropical Bioresources, Institute of Tropical Agriculture and Forestry, Hainan University, Haikou 570228, China

[3] School of Computing, Clemson University, Clemson, 29634-0974, USA

# These authors contributed equally to the manuscript as first authors.

* To whom correspondence should be addressed:

Feng Luo: Email: luofeng@clemson.edu

Liang-Ying Dai: Email: daily@hunau.net

Jun Tao: Email: taoj@hainu.edu.cn

Table S1. The datasets of seven Xoc strains in this study

Strain	Sample ID	SMRT cell	Total length of subreads (Gb)	Coverage	Reference link
BLS256	SAMN03612250	5	1.94	421x	https://www.ncbi.nlm.nih.gov/genome/529?genome_assembly_id=233712
BLS279	SAMN03612248	4	1.66	362x	https://www.ncbi.nlm.nih.gov/genome/529?genome_assembly_id=233507
CFBP2286	SAMN03612252	6	2.03	425x	https://www.ncbi.nlm.nih.gov/genome/529?genome_assembly_id=233505
CFBP7331	SAMN03612260	7	2.11	442x	https://www.ncbi.nlm.nih.gov/genome/529?genome_assembly_id=233509
CFBP7341	SAMN03612287	4	1.85	387x	https://www.ncbi.nlm.nih.gov/genome/529?genome_assembly_id=233510
L8	SAMN03612258	4	1.33	291x	https://www.ncbi.nlm.nih.gov/genome/529?genome_assembly_id=233511
RS105	SAMN03612254	4	1.80	395x	https://www.ncbi.nlm.nih.gov/genome/529?genome_assembly_id=233512

Table S2. The stop codon usage in seven strains

Strain	Normal stop codon			6mA stop codon		
	TGA	TAA	TAG	TGA	TAA	TAG
BLS256	2681 (67.63%)	817 (20.61%)	466 (11.76%)	56 (49.56%)	47 (41.59%)	10 (8.85%)
BLS279	2697 (68.19%)	805 (20.35%)	453 (11.46%)	66 (51.56%)	49 (38.28%)	13 (10.16%)
CFBP2286	2807 (67.51%)	860 (20.68%)	491 (11.81%)	80 (60.15%)	42 (31.58%)	11 (8.27%)
CFBP7331	2762 (67.28%)	866 (21.10%)	477 (11.62%)	73 (50.69%)	59 (40.97%)	12 (8.34%)
CFBP7341	2803 (67.32%)	874 (20.98%)	487 (11.70%)	45 (47.37%)	47 (49.47%)	3 (3.16%)
L8	2714 (67.85%)	831 (20.78%)	455 (11.37%)	42 (55.26%)	26 (34.21%)	8 (10.53%)
RS105	2696 (68.20%)	807 (20.42%)	450 (11.38%)	59 (48.76%)	49 (40.50%)	13 (10.74%)

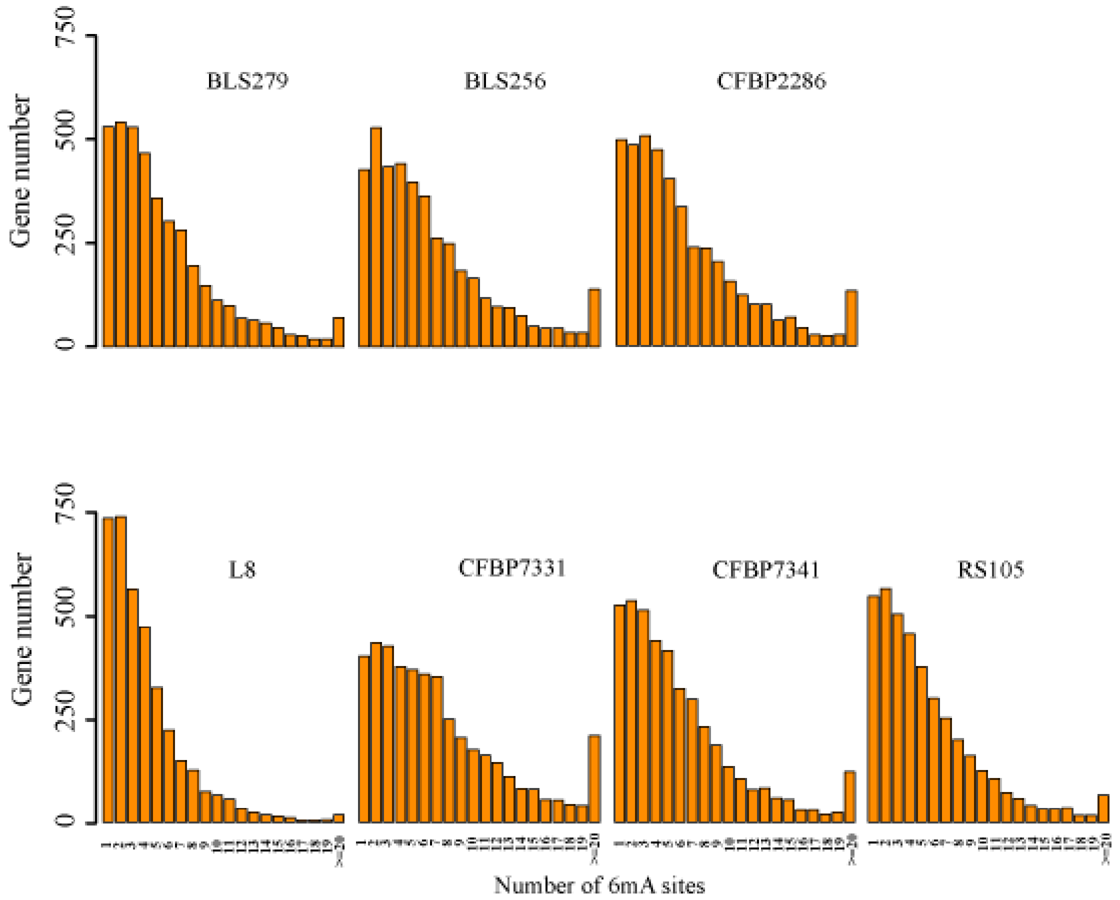

Figure S1. The located 6mA sites number of all genes in seven strains.

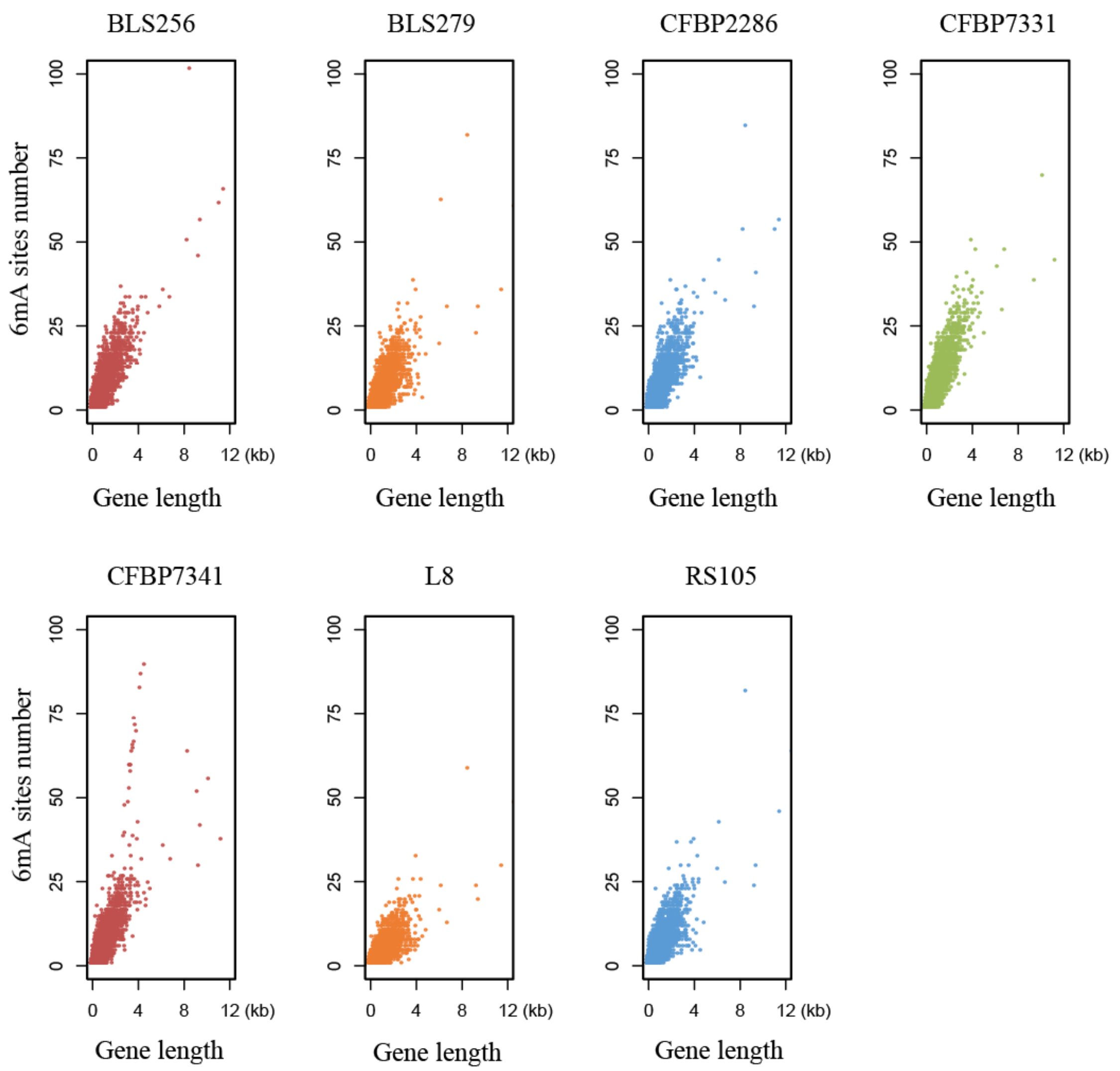

Figure S2. The scatter plot of 6mA sites numbers and gene length in seven strains.

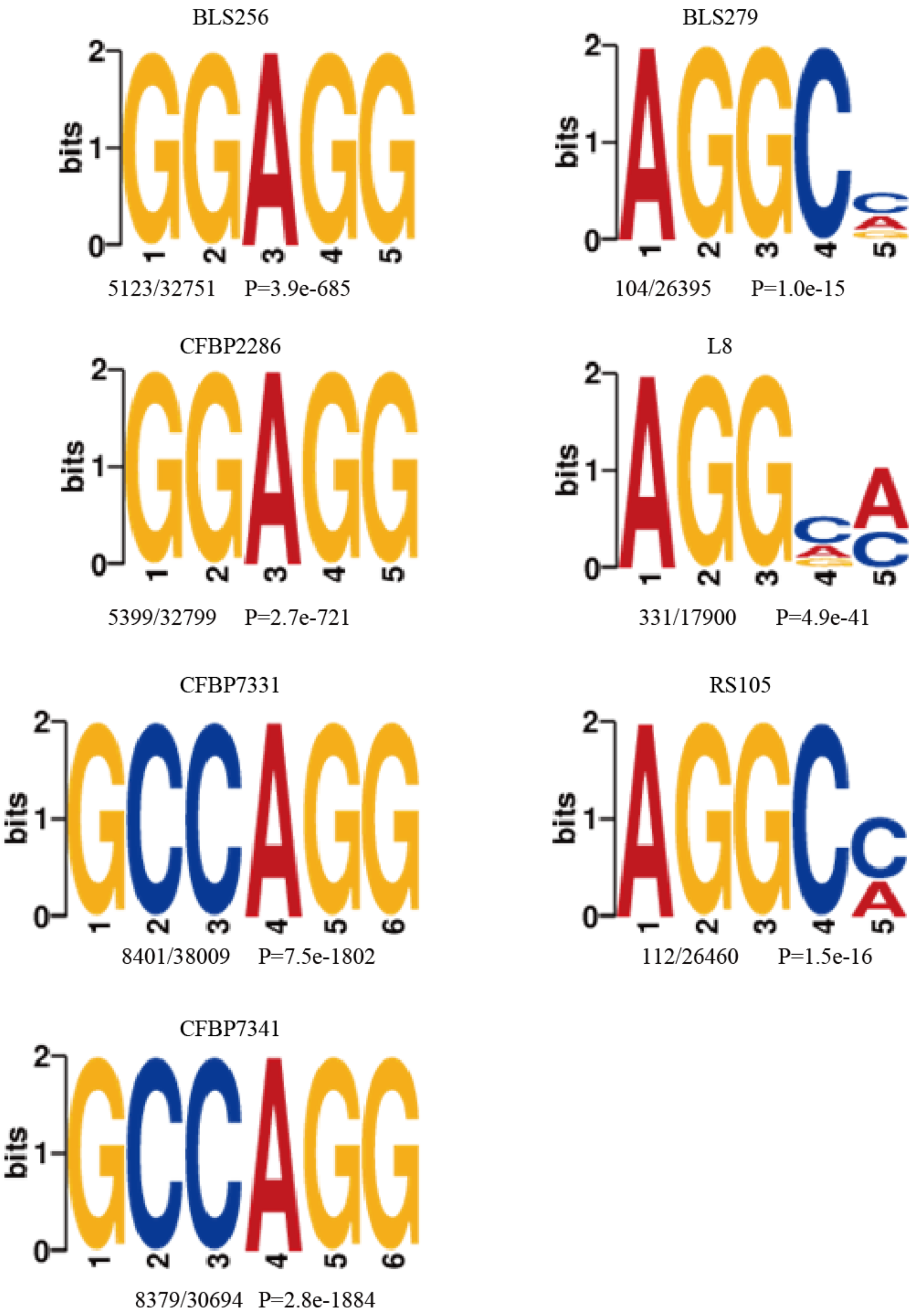

Figure S3. The identified consensus motifs containing 6mA sites in seven strains.

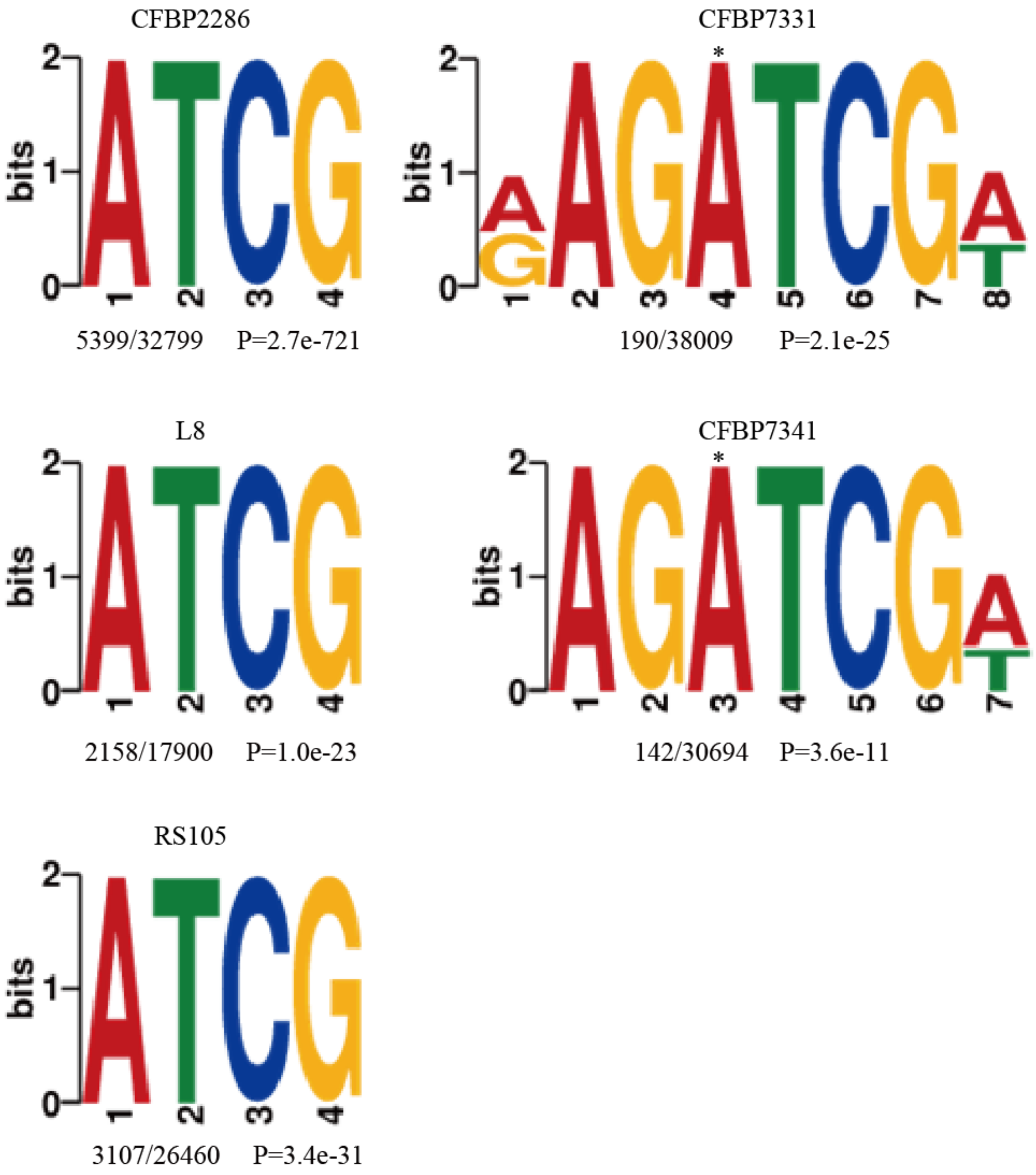

Figure S4. The identified ATCG motif containing 6mA sites in partial strains.
